# *Toxoplasma gondii* exposure in Brazilian indigenous populations, their dogs, environment, and healthcare professionals

**DOI:** 10.1016/j.onehlt.2023.100567

**Published:** 2023-05-18

**Authors:** Fernando Rodrigo Doline, João Henrique Farinhas, Leandro Meneguelli Biondo, Pollyanne Raysa Fernandes de Oliveira, Nássarah Jabur Lot Rodrigues, Karina Pavão Patrício, Rinaldo Aparecido Mota, Helio Langoni, Christina Pettan-Brewer, Rogério Giuffrida, Vamilton Alvares Santarém, Wagner Antônio Chiba de Castro, Andrea Pires dos Santos, Louise Bach Kmetiuk, Alexander Welker Biondo

**Affiliations:** a Graduate College of Cellular and Molecular Biology, Federal University of Paraná, Curitiba, PR, Brazil; b National Institute of the Atlantic Forest (INMA), Brazilian Ministry of Science, Technology, and Innovation, Santa Teresa, Espirito Santo, Brazil; c Graduate College of Animal Bioscience, Federal Rural University of Pernambuco, Recife, PE, Brazil; d Department for Animal Production and Preventive Veterinary Medicine department for Animal Production and Preventive Veterinary Medicine, Botucatu, SP, Brazil; e Department of Public Health, Medical School, São Paulo State University, Botucatu, SP, Brazil; f Department of Comparative Medicine, School of Medicine, University of Washington, Seattle, WA, USA; g Laboratory of Veterinary Parasitology, Veterinary Teaching Hospital, University of Western São Paulo, Presidente Prudente, SP, Brazil; h Latin-American Institute of Life and Nature Sciences, Federal University for Latin American Integration, Foz do Iguaçu, PR, Brazil; i Department of Comparative Pathobiology, Purdue University, West Lafayette, IN, USA

**Keywords:** Health inequities, Public health, Water quality

## Abstract

Although *Toxoplasma gondii* exposure has been reported in indigenous populations worldwide, a One Health approach has not been applied to date. This study concurrently assessed *T. gondii* exposure in indigenous populations, and their dogs, environment, and indigenous or non-indigenous healthcare professionals (HPs). Human and dog serum samples from 9 indigenous communities in Brazil were assessed by indirect immunofluorescence antibody test for anti-*T. gondii* antibodies. Soil samples (30 per community) were processed with PCR to amplify *T. gondii* DNA. Associated risk factors and seroprevalence were analyzed using logistic regression models. Human seropositivity and type of water source were assessed by generalized linear mixed model (GLMM) with binomial error distribution, and game meat consumption with chi-squared test. Overall, 225/463 (49%) indigenous persons were seropositive for anti-*T. gondii* antibodies. Of all the HPs, 67/168 (40%) were positive, and included 54/147 (37%) positive non-indigenous HPs. Indigenous persons more likely to be seropositive compared with non-indigenous HPs (OR: 1.63; 95% CI: 1.11–2.39). A total of 97/253 (38%) dogs were seropositive and highly associated with seropositive owners (*p* < 0.001). Based on univariate analysis for indigenous individuals, state location of community (p < 0.001), ethnicity (p < 0.001), consumption of game meat (p < 0.001), type of water source (*p* < 0.001), and educational level (*p* = 0.026) were associated with seropositivity. Logistic regression showed that indigenous seropositivity was associated with eating game meat (*p* = 0.002), drinking water from rivers (*p* < 0.001), and inversely proportional to the educational level. According to univariate analysis for non-indigenous HP, age (*p* = 0.005), frequency of visits to the indigenous populations (*p* < 0.001), consumption of water at the indigenous communities (p < 0.001), and ingestion of raw meat (*p* = 0.023) were associated with *T. gondii* seropositivity. Logistic regression revealed living outdoors (*p* = 0.042), habit of hunting (*p* = 0.008), and drinking river water (*p* = 0.007) as risk factors associated to seropositivity in dogs. In addition, indigenous communities lacking water treatment had higher seroprevalence for all groups including indigenous persons (GLMM; z = −7.153; *p* < 0.001), their dogs (GLMM; z = −2.405; *p* = 0.0162), and all HPs (GLMM; z = −2.420; *p* = 0.0155). Human seropositivity was associated with that of their dogs (p < 0.001). A single soil sample, out of 270 (0.37%), was positive for *T. gondii* by PCR. Our results indicate water source is a risk for human and dog toxoplasmosis in indigenous communities; both share similar exposure. Moreover, quality water access was shown to be crucial to prevent toxoplasmosis in both total and non-indigenous HPs who work in these indigenous communities.

## 1 Introduction

The self-identified indigenous population in Brazil, based on the 2010 Brazilian Census, was estimated at 896,000 individuals, approximately 0.4% of the total population. They are distributed across 505 lands (12.5% of Brazilian territory), have 305 ethnicities, and 274 spoken languages. The population has a large proportion of young persons (46% are under 20 years of age) and they represent one of the highest levels of socio-diversity worldwide [1]. Brazilian indigenous populations have faced injustices since colonization, which negatively impacts their cultures and promotes exclusion and isolation [2]. In addition, such native populations were settled in remote geographic areas lacking basic sanitation, potable tap water, and veterinary assistance [3]. Consequently, indigenous populations harbor a higher prevalence of zoonoses when compared to other Brazilian ethnicities [4].

Toxoplasmosis is an anthroponotic protozoan disease caused by *Toxoplasma gondii*, an obligate intracellular coccidia, of the phylum Apicomplexa that frequently infects homeothermic species (mammals, birds) worldwide [5]. Despite felids possibly serving as a direct source of infection, toxoplasmosis has been classified as a foodborne disease, mostly transmitted by ingestion of contaminated water, soil, and food (especially raw or undercooked meat) [6]. In this scenario, toxoplasmosis requires a One Health approach; defined as a systemic and integrated approach to ecosystems involving humans, animals, and environmental health [7].

Approximately one-third of worldwide population may be already infected with *T. gondii* [8]. Prevalence ranges from 1% to 78%, with higher rates occurring in Latin American countries [9]. In Brazil, 25 outbreaks (including the 2 largest worldwide outbreaks to date) were reported in the past 50 years, with nearly one-half between 2010 and 2018. Most outbreaks were due to oocyst contamination of water, fruits, and vegetables [8]. Serosurveys of *T. gondii* have been conducted in Brazilian indigenous populations, with prevalence varying by community. Overall, indigenous populations have toxoplasmosis seropositivity ranging from 26% to 100% (mean = 63%, SD 21.7%; 95% CI: 21.2–96.5) [[10], [11], [12], [13], [14], [15], [16], [17], [18], [19]] (Supplementary Table 1).

Despite indigenous populations being considered vulnerable to several infectious diseases, particularly those which are waterborne and attributed to inadequate water supply system, storage, and access to potable water [20], concurrent surveys of human, animal, and environmental seropositivity or molecular presence of *T. gondii* are scarce. This study aimed to apply a One Health approach to assess and compare the anti-*T. gondii* antibody presence in indigenous persons, their dogs, and healthcare professionals (HPs), along with a molecular survey of soil samples in indigenous communities in southern and southeastern Brazil.

## 2 Material and methods

### 2.1 Ethics statement

This study was approved by three different indigenous organizations which were submitted together and approved by the Ethics Committee in Human Health of the Brazilian Ministry of Health (protocol 52,039,021.9.0000.0102) and by the Ethics Committee of Animal Use (protocol number 033/2021) of the Federal University of Paraná.

### 2.2 Study design

This was a cross-sectional epidemiological study with a One Health approach describing human and dog populations living in or visiting indigenous communities in the states of Paraná and São Paulo, southern Brazil, conducted from December 2020 through February 2022.

### 2.3 Study areas

The study was conducted in nine different indigenous communities of Guarani, Terena, and Kaingang ethnicities in the states of Paraná and São Paulo (Supplementary Table 2). The five indigenous communities of Paraná state were located within the Atlantic Forest biome areas, while the four in São Paulo state were within the Cerrado biome [21].

### 2.4 Indigenous communities

In the five communities of Paraná state, the indigenous communities have strong environmental connections, relying mostly on natural resources for living such as wildlife hunting, fishing, and subsistence agriculture [22]. Craftspersonsuse natural materials to provide secondary income from items such as baskets, necklaces, miniature wildlife animals carved in wood, spears, bows, and arrows [22,23]. These five indigenous communities, located in isolated areas, lacked basic sanitary systems and tap water treatment. For example, the *Kuaray haxa* indigenous community was on a preserved oceanic island accessed only by boat. In the four indigenous communities of São Paulo state, agriculture was the main economic activity, producing crops for community consumption and outside trading [24]. Makers also contributed to subsistence [25], and some indigenous persons worked outside the community at nearby rural areas or in urban commerce [26]. These four communities were not as isolated as those in Paraná and they had basic sanitary systems and access to artesian well water. Indigenous communities were grouped by sanitary system (adequate or not), water source (treated or not) and feces destination (environment, pit latrine, septic tank).

### 2.5 Healthcare professionals

Along with the indigenous populations, HPs working in the communities were also sampled. Their samples were taken during expeditions and specific visits to the Special Department of Indigenous Health (SDIH) Headquarters called Seashore South, one of the 34 nationwide divisions of the Special Secretary of Indigenous Health, Brazilian Ministry of Health. The SDIH – Seashore South was managing 25,784 indigenous persons with 25 ethnicities, living within 174,521.07 km^2^ (twice the size of Portugal) and having 129 indigenous communities located in four states: Santa Catarina, Paraná, São Paulo, and Rio de Janeiro [26].

Healthcare professionals were categorized into three groups according to their function, level, and frequency of visits to the indigenous populations. The group of high-level contact professionals included physicians, nurses, nursing technicians, drivers, and teachers that often visited the communities; medium-level contact professionals included the multi-disciplinary SDIH teams that periodically visit the communities; and low-level contact professionals, included administration and health professionals having sporadic visits to indigenous communities.

### 2.6 Samplings

#### 2.6.1 Blood samples

Human participants, both indigenous persons and HPs, were sampled following signed consent and completion of a specific epidemiological questionnaire. Human blood samples were collected with cephalic venipuncture by certified nurses. Dog blood samples were collected with jugular venipuncture by certified veterinarians. Blood samples placed in sterile tubes containing ethylenediaminetetraacetic acid (EDTA), for Measure packed cell volume (PCV) and total plasma protein concentration (TPP). Samples were also collected in tubes without anti-coagulant and kept at room temperature (25 °C) until visible clot retraction, then centrifuged at 800*g* for five minutes, and serum separated and kept at −20 °C until processing.

#### 2.6.2 Soil samples

Soil samples were randomly collected from each indigenous community around the school (*n* = 10), healthcare service (n = 10), and recreation areas such as soccer or gathering fields (n = 10). At the time of sampling, characteristics of each site were recorded including, local geographic coordinates, presence of children and adults having direct contact with soil, presence of dogs and cats, and presence of feces. Approximately 100 g of soil at a depth of 5 to 10 cm was collected, as previously established [27], placed into a plastic bag, and stored at 4 °C until processing.

### 2.7 Epidemiological data

Epidemiological data was obtained by interviews using questionnaires at the time of blood sampling, and with the use of an indigenous translator when necessary. Questions included personal information such as age, sex, education level attained, community and duration of residence, ethnicity, occupation, hunting habit and frequency, owned animals, drinking water source, habit of washing fruits and vegetables, washing hands prior to meals, and consumption of raw or undercooked meat. Participants owning dogs signed consent for animal sampling and provided information about the dog's age, sex, breed, origin (purchased, given, adopted), location of residence, diet, consumption of raw meat, drinking water source, access to forest, hunting habit, ectoparasite and endoparasite presence and control, and vaccinations.

Our study assumed veterinary intervention as a component of ethical research. All sampled (and non-sampled voluntarily brought) dogs were examined. And, the following were given: rabies vaccine (Immunovet R, Biovet, São Paulo, SP, Brazil); species-specific vaccines against distemper, hepatitis, adenovirus, parainfluenza, parvovirus, coronavirus, *Leptospira* serovars Canicola, Icterohaemorrhagiae, Copenhageni and Grippotyphosa (Poly 10, Lema-Injex Biologic, Lagoa Santa, MG, Brazil); oral antiworm treatment (Vermivet, Biovet, São Paulo, SP, Brazil); pour-on treatment for ticks and fleas (Topline, Boehringer-Ingelheim, Campinas, SP, Brazil); and treatment for sarcoptic mange (scabies) and tungiasis (Ivomec Gold, Boehringer-Ingelheim, Campinas, SP, Brazil). All products were purchased from nearby certified veterinary drugstores with long expiration dates; vaccines were kept cool with recyclable ice until application. Owners were given a cell-phone contact in case of dog immunoreaction or side-effects. In addition, vinyl banners against dog and cat abandonment were printed with research funds and sent for posting at the entry of each of 96 indigenous communities located in the Paraná state.

### 2.8 Laboratorial analysis

#### 2.8.1 Serological test

Detection of anti-*T. gondii* antibodies was performed with the indirect immunofluorescence reaction (IFR) test [27]. Human serum samples were tested for IgM and IgG class antibodies against *T. gondii*, and canine serum samples were tested solely for anti-*T. gondii* IgG class antibodies. Serial dilutions of 1:16, 1:64, 1:256, 1:1024, and 1:4096 were made with phosphate buffered saline (PBS) pH 7.2. Immunofluorescence slides were sensitized with 0.1% formaldehyde to inactivated *T. gondii* tachyzoites (RH strain) obtained from an intraperitoneal wash in Swiss mice after 3 days of inoculation. Secondary antibodies were conjugated with fluorescein isothiocyanate (Bethyl-Montgomery, TX, USA) for fluorescence. Commercial human-specific anti-IgM and anti-IgG antibodies and dog-specific anti-IgG antibodies were used. Samples were considered positive if antibody titers were greater than or equal to 16 (the established cutoff point). For seropositive samples, final titers were determined as the last dilution at which ≥50% fluorescence remained at the border of the tachyzoites.

#### 2.8.2 Recovery of *T. gondii* oocysts from soil samples

For each sample collected, subsamples of 10 g were used in duplicate and 5 mL of 2% sulfuric acid was added to each subsample, followed by a 16 h rest to allow sporulation to occur [28]. After sporulation, the first stage of oocyte recovery began with the addition of 30 mL of 1 M glycine, homogenization for 30 min, and a 5-min rest for sedimentation [28]. The recovered supernatant was centrifuged at 1500*g* for 15 min and formed a pellet. In the second step, the pellet was treated with an established technique [28], and a 2 mL aliquot of the final supernatant was distributed into two microtubes, followed by the addition of 1 mL of ultrapure water solution and centrifugation at 1500*g* for 5 min. To a volume of 200 μL of pellet from the previous step, 1 mL of Tris-HCl, NaCl and EDTA (TE) buffer was added and centrifuged as above. A final volume of 200 μL of reconstituted pellet from each duplicate was stored in a single microtube.

#### 2.8.3 Oocyst DNA extraction

After the recovery process and addition of the lysis buffer (T1 buffer, NucleoSpin Tissue kit - Macherey-Nagel, Düren, Germany) the samples were submitted to 10 freeze-thaw cycles carried out in liquid nitrogen and a water bath adjusted to 56 °C [29]. DNA was extracted from soil samples using a commercial kit (NucleoSpin Tissue, Macherey-Nagel, Düren, Germany) according to the manufacturer's instructions, with the following modifications: samples were incubated in lysis buffer (T1) and proteinase K at 56 °C for 16 h [29].

#### 2.8.4 Nested-PCR for Apicomplexa

For n-PCR of 18S rDNA, the external primers were Tg18s48F, CCATGCATGTCTAAGTATAAGC and Tg18s359R, GTTACCCGTCACTGCCAC, which amplify a 312 bp DNA fragment from T. gondii. The nested PCR primers were Tg18s58F, CTAAGTATAAGCTTTTATACGGC and Tg18s348R, TGCCACGGTAGTCCAATAC, which amplify a 291 bp DNA fragment from *T. gondii*, as previously established [31]. The thermal profile included initial denaturation at 94 °C for 2 min, 30 cycles of denaturation at 94 °C for 30 s, annealing at 57 °C for 30 s, extension at 72 °C for 30 s, and a final extension at 72 °C for 1 min, for the first and second reaction, with only a change in the number of cycles in the last one, which was 35 cycles. The reaction mix consisted of 0.5 μL of each 2 μM primer, 6.25 μL of a commercial PCR master mix (GoTaqGreen, Promega, Madison, WI, USA), 2.75 μL of ultrapure water, and 2.5 μL of DNA in a final volume of 12.5 μL [32]. The amplified product of approximately 300 base pairs was detected by electrophoresis using 2% agarose gel, stained with Blue Green (LGC Biotechnology), and subsequently photo-documented (Loccus - L-PIX EX).

#### 2.8.5 Sequence analysis

Positive amplicons for the 18S rDNA gene were purified using a commercial extraction kit (QIAquick Gel Extraction Kit, Qiagen, Valencia, CA, USA) as described by the manufacturer. Purified products were sequenced bidirectionally using a commercial sequencing Kit (BigDye Terminator v3.1 Cycle, Applied Biosystems, Foster City, CA), following the manufacturer's instructions. The sequencing reaction was performed by capillary electrophoretic separation on a commercial sequencer (ABI 3500 Genetic Analyzer, Applied Biosystems, Foster City, CA). Sequence analysis was performed using a commercially available software (Staden Package 4.1.4, Gene Codes Corporation, USA), and all sequences were compared with the Basic Alignment Search Tools of the National Center for Biotechnology Information (www.ncbi.nlm.nih.gov) to identify the species.

### 2.9 Statistical analysis

The initial approach to associated risk factors and *T. gondii* seroprevalence was assessed by a logistic regression model. Seroprevalence for *T. gondii* antibodies (IgG) was estimated for each studied group: a) the communities indigenous persons; b) HPs; and c) dogs owned by indigenous persons. As indigenous HPs may have been a potential confounding factor to estimating the risk factor among the healthcare professional group, only the non-indigenous HPs were included in both univariate and logistic regressions. Initially, univariate analysis was performed by chi-square test. Variables with *P* values <0.2 in univariate analysis were considered fit to build a multiple regression model. Akaike's information criterion was used as the stopping rule. Highly correlated predictors were removed from the logistic model to prevent problems of multicollinearity. The odds ratio (OR) and 95% confidence interval (CI) were calculated for each variable. *P* values <0.05 were considered statistically significant. The predictive power of the models was presented as area under curve (AUC) of the receiver operating characteristic curve (ROC curve). R version 4.2.2 software (R Project for Statistical Computing) was used to run the statistical analysis. Association between seropositivity in the indigenous persons and their dogs was reached by the chi-square test. Data of indigenous communities with water treatment were used to assess the potential association between hunting meat consumption and seropositivity of indigenous communities using chi-squared test. Data from indigenous communities with water treatment were used for assessment between seropositivity of dogs and owners, using the Fisher's exact test. All models were conducted in the computational R Project [33].

In addition, the effect of water treatment over the human and dog seropositivity at the indigenous communities was assessed by generalized linear mixed model (GLMM) with binomial error distribution [34]. In the models, the fixed effect included the absence or presence of water treatment in each indigenous community, and response variable was the absence or presence of seropositivity in individuals of populations. The water treatment effect, disposition of water consumption in indigenous communities, and interaction of these two factors over the seropositivity of HPs was assessed using a GLMM with negative binomial error distribution, by the package lme4, function glmer.nb. In the model, the fixed effect included the absence or presence of water treatment in the group of indigenous communities of which these professionals often worked at the time, as well as the disposition or not of water consumption at the indigenous communities by these professionals, with the response variable was the absence or presence of individual seropositivity on HPs.

## 3 Results

**Fig. 1:**
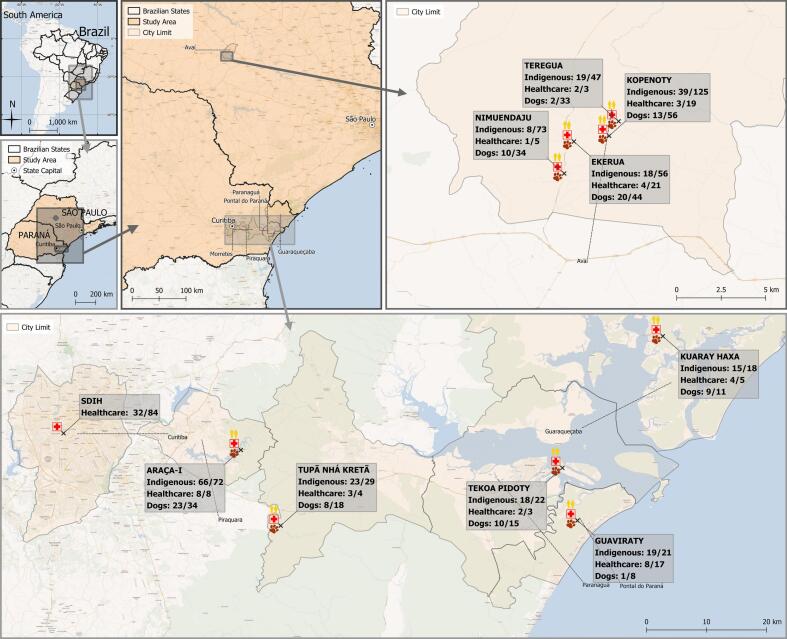
Sampling location and anti-*T. gondii* antibodies for the indigenous communities, including indigenous populations, healthcare professionals and soil samples (single PCR positive) in the states of Paraná and São Paulo.

The study included a total of 463 indigenous persons living in the communities and 168 healthcare professionals that visited. Of the healthcare professionals, 147 were non-indigenous and 21 were indigenous. Seroprevalence of indigenous persons living the communities was observed in 225/463 (49%; 95% CI: 44.1–53.1), while HPs had 3/168 (2%) IgM (titers of 16) and 67/168 (40%) IgG (titers of 16 to 1024) anti-*T. gondii* antibodies, including 54/147 (37%; 95% CI: 29.4–44.8) of the non-indigenous HPs. Indigenous persons had increased odds of being seropositive for anti-*T. gondii* IgG compared with non-indigenous HP (OR: 1.63; 95% CI: 1.11–2.39). Likewise, indigenous HPs (*n* = 21) were 2.8 more likely (95% CI: 1.08–7.48) to be seropositive when compared to non-indigenous HP (*n* = 147) (Supplementary Table 3). In dogs, seroprevalence was of 97/253 (38%; 95% CI: 32.5–44.5) positive animals. Increased risk of seropositivity in dogs was highly associated (OR = 37.7; 95% CI = 18.5–82.9; *p* < 0.001) with the presence of a seropositive owner (Fig. 1).

Overall, indigenous persons presented 11/463 (2%) IgM (titers of 16) and 225/463 (49%) IgG (titers of 16 to 1024) anti-*T. gondii* antibodies, while HPs presented 3/168 (2%) IgM (titers of 16) and 67/168 (40%) IgG (titers of 16 to 1024) anti-*T. gondii* antibodies (Supplementary Table 3; Supplementary Table 4; Supplementary Table 5). The seropositivity in indigenous communities with no water treatment was 10/162 (6%) for IgM and 141/162 (87%) for IgG, while in indigenous communities with water treatment seropositivity was 1/301 (0.3%) for IgM and 84/301 (28%) for IgG anti-*T. gondii* antibodies (Supplementary Table 6).

According to univariate analysis for indigenous populations, state location of the community (*p* < 0.01), ethnicity (*p* < 0.0001), consumption of game meat (p < 0.0001), drinking water source (p < 0.0001), and educational level (*p* = 0.026) were associated with *T. gondii* seropositivity. Associated risk factors were not statistically significant for indigenous persons regarding gender (*p* = 0.981), age (*p* = 0.066), consumption of raw meat (*p* = 0.185), or being a cat owner (*p* = 0.929) (Table 1).

**Table 1:** Associated risk factors for anti-*Toxoplasma gondii* antibodies (IgG) in the indigenous population in south (Paraná state) and southeast (São Paulo state) Brazil (*N* = 463), by univariate and multivariate statistical analysis.

	ELISA test result		Univariate analysis		Multivariate analysis	
	Positive (%)	Negative (%)	OR (CI 95%)	*p*-value	OR (CI 95%)	*p*-value
Variables	225 (48.6)	238 (51.4)				
Location				<0.001		
São Paulo	84 (37.3)	217 (91.2)	1 [Reference]			
Paraná	141 (62.7)	21 (8.82)	17.1 (10.3–29.6)			
Gender				0.981		
Female	116 (51.6)	124 (52.1)	1 [Reference]			
Male	109 (48.4)	114 (47.9)	1.02 (0.71–1.47)			
Age				0.066		
3–17	56 (24.9)	81 (34.0)	1 [Reference]			
18–26	48 (21.3)	50 (21.0)	1.39 (0.82–2.35)			
27–40	67 (29.8)	49 (20.6)	1.97 (1.19–3.27)			
41–89	54 (24.0)	58 (24.4)	1.34 (0.81–2.23)			
Ethnicity				<0.001		
Guarani	156 (69.3)	89 (37.4)	Ref.			
Kaigang	5 (2.22)	6 (2.52)	0.48 (0.13–1.68)			
Terena	64 (28.4)	143 (60.1)	0.26 (0.17–0.38)			
Educational level				0.026		
Illiterate	12 (5.33)	1 (0.42)	Ref.		1 [Reference]	
Primary	29 (12.9)	33 (13.9)	0.08 (0.00–0.48)		0.05 (0.00–0.31)	0.007
Elementary	83 (36.9)	101 (42.4)	0.08 (0.00–0.41)		0.06 (0.00–0.32)	0.008
High	79 (35.1)	82 (34.5)	0.09 (0.00–0.49)		0.06 (0.00–0.34)	0.009
Graduate	22 (9.78)	21 (8.82)	0.10 (0.00–0.59)		0.08 (0.00–0.48)	0.022
Meat consumption				0.185		
Well-done	217 (96.4)	222 (93.3)	1 [Reference]		1 [Reference]	
Raw/undercooked	8 (3.56)	16 (6.72)	0.52 (0.20–1.21)		0.44 (0.15–1.17)	0.116
Consumption of game meat				<0.001		
No	74 (32.9)	139 (58.4)	1 [Reference]		1 [Reference]	
Yes	151 (67.1)	99 (41.6)	2.86 (1.96–4.19)		2.0 (1.29–3.01)	0.002
Drinking water source				<0.001		
Spring/artesian well	136 (60.4)	226 (95.0)	1 [Reference]		1 [Reference]	
River	89 (39.6)	12 (5.04)	12.1 (6.63–24.2)		10.88 (5.86–21.90)	<0.000 1
Cat owner				0.929		
No	121 (53.8)	130 (54.6)	1 [Reference]			
Yes	104 (46.2)	108 (45.4)	1.03 (0.72–1.49)			

Despite the finding that indigenous persons living in the Paraná state were 17 times more likely to be seropositive when compared to indigenous persons São Paulo state, state location was not considered appropriate for inclusion in the logistic regression due to possible collinearity with ethnicity. Logistic regression (backward stepwise using two steps) revealed the following information: 1) odds of higher seropositivity was associated with lower educational level; 2) odds of seropositivity was associated with game meat consumption (OR: 2.0; 95% CI: 1.29–3.01; *p* = 0.0016) and with drinking water from river (OR: 10.9; 95% CI: 5.86–21.9; *p* < 0.0001). The discriminative power of the final logistic regression model was considered moderate (AUC = 0.75; 95% CI: 0.70–0.79).

According to univariate analysis for non-indigenous HPs, age (*p* = 0.005), frequency of visits to the indigenous populations (p < 0.0001), consumption of water at the indigenous communities (p < 0.0001), and ingestion of raw meat (*p* = 0.023) were associated with *T. gondii* seropositivity. On the other hand, gender (*p* = 0.752), ethnicity (*p* = 0.760), having piped/treated water at home (*p* = 1.0), washing vegetables before meal (p = 1,0), consumption of game meat (*p* = 0.289), and having meals at communities (*p* = 0.625) showed no association to the presence of anti-*T. gondii* antibodies (Supplementary Table 7). As already mentioned, indigenous HPs represented a potential confounding factor to estimate the risk factor among healthcare professional group, and thus only the group of non-indigenous HPs was included in both univariate and logistic regressions.

In non-indigenous HPs, higher working frequency (OR: 4.74; 95% CI: 1.17–24.58; *p* = 0.04), higher frequency of water consumption at the communities (OR: 27.0; 95% CI: 8.86–104.23; *p* < 0.0001), and ingestion of raw or undercooked meat (OR: 5.56; 95% CI: 1.49–21.5; *p* = 0.011) were associated with higher seropositivity. The logistic regression model showed a discriminative power between moderate to excellent (AUC = 0.88; 95% CI: 0.81–0.94).

The logistic regression model for dogs showed that drinking water source (*p* = 0.00072), habit of hunting (*p* = 0.0068), and living outdoors (*p* = 0.046) were variable associated with the presence of *T. gondii* antibodies (IgG), while sex (*p* = 0.334) and food with raw meat (*p* = 0.107) were not associated according to the univariate analysis (Table 2). Seropositivity likelihood was approximately 4-fold higher in dogs living in the Paraná than São Paulo state. However, due to the observation of collinearity, location (state) was not considered to the multivariate analysis. Logistic regression (using the backward stepwise) revealed living outdoors (OR: 3.84; 95% CI: 1.19–17.33; *p* = 0.042), hunting habit (OR: 2.77; 95% CI: 1.32–5.97; *p* = 0.0077), and drinking river water (OR: 2.44; 95% CI: 1.27–4.74; *p* = 0.0074) as associated risk factors for dog seropositivity. The final logistic model was considered to have a moderate discriminative power (AUC = 0.68; 95% CI: 0.62–0.74).

**Table 2:** Associated risk factors for anti-*Toxoplasma gondii* antibodies (IgG) in dogs in dogs living at indigenous communities in south (Paraná state) and southeast (São Paulo state) Brazil (*N* = 253), by univariate and multivariate statistical analysis.

	ELISA test result		Univariate analysis		Multivariate analysis	
	Positive (%)	Negative (%)	OR (CI 95%)	*p*-value	OR (CI 95%)	*p*-value
Variables	97 (38.3)	156 (61.7)				
Location				<0.001		
São Paulo	45 (46.4)	122 (78.2)	1 [Reference]			
Paraná	52 (53.6)	34 (21.8)	4.11 (2.38–7.21)			
Gender				1.0		
Female	49 (50.5)	78 (50.0)	1 [Reference]			
Male	48 (49.5)	78 (50.0)	0.98 (0.59–1.63)			
Age (months)				0.334		
≤12	22 (22.7)	45 (28.8)	1 [Reference]			
12–48	60 (61.9)	95 (60.9)	1.29 (0.71–2.39)			
≥49	15 (15.5)	16 (10.3)	1.90 (0.79–4.61)			
Living outdoors				0.046		
No	3 (3.09)	17 (10.9)	1 [Reference]		1 [Reference]	
Yes	94 (96.9)	139 (89.1)	3.67 (1.18–16.7)		3.84 (1.19–17.33)	0.042
Drinking water source				0.001		
River	66 (68.0)	135 (86.5)	1 [Reference]		1 [Reference]	
Spring/artesian well	31 (32.0)	21 (13.5)	3.00 (1.61–5.70)		2.44 (1.27–4.74)	0.007
Raw meat consumption				0.107		
No	6 (6.19)	21 (13.5)	1 [Reference]		1 [Reference]	
Yes	91 (93.8)	135 (86.5)	2.31 (0.94–6.60)		2.66 (1.05–7.66)	0.050
Hunting habit				0.007		
No	74 (76.3)	140 (89.7)	1 [Reference]		1 [Reference]	
Yes	23 (23.7)	16 (10.3)	2.70 (1.35–5.54)		2.77 (1.32–5.97)	0.008

Overall, indigenous community populations without water treatment had statistically higher seroprevalence when compared to populations living with water treatment, for both persons (GLMM; z = −7.153; *p* < 0.001) and dogs (GLMM; z = −2.405; *p* = 0.0162) (Table 3; Fig. 1A).

**Table 3:** Generalized Linear Mixed Model (GLMM) for *T. gondii* seropositivity of indigenous populations, their dogs, and healthcare professionals in southern/southwestern Brazil associated with consumption of drinkable water.

Group	Treatment	Estimate	Std error	z value	P
Indigenous	Intercept	1.9970	0.3233	6.1773	<0.001*
	Treatment water	−2.9767	0.4162	−7.153	<0.001*
Dogs	Intercept	0.3613	0.4452	0.812	0.4170
	Treatment water	−1.5276	0.6352	−2.405	0.0162*
Health professionals	Intercept	−0.9445	0.3544	−2.665	0.0077*
	Treatment water	−15.8615	6.5541	−2.420	0.0155*
	Drink water in the communities	0.8873	0.4228	2.099	0.0358*
	Do not drink water in indigenous communities lacking water treatment	15.2255	6.5680	2.318	0.0204*

The HPs that worked in indigenous communities with water treatment had significantly lower seropositivity than those working in communities without water treatment (GLMM; z = −2.420; *p* = 0.0155). In addition, the HPs who drank water at the indigenous communities presented higher seropositivity when compared to those who did not (GLMM; z = 2.099; *p* = 0.0358). Finally, even professionals that did not drink water in indigenous communities lacking water treatment had higher seropositivity than those not consuming water in indigenous communities with water treatment (GLMM; z = 2.318; *p* = 0.0204) (Fig. 1B).

The seropositivity of indigenous persons owning dogs was associated with their dogs seropositivity (OR = 25.88; IC = 95%; Fisher exact test <0.001). Consumption of game meat by indigenous persons was not a risk factor for *T. gondii* exposure (OR = 1.09; IC = 95%;*p* = 0.75).

Only 1 soil sample of 270 (0.4%) was positive to gene 18S rDNA of Apicomplexa parasites It was obtained at the indigenous community of Araça'í (3%; 1/30, which lacked water treatment (Supplementary Table 8). This sample presented 100% similarity to *T. gondii* gene 18rDNA sequences (KX 008024.1 to KX008033.1). The *T. gondii* genotyping was unsuccessful due to the insufficient amount of DNA recovered from the original sample.

## 4 Discussion

This study represents the first practical One Health approach to investigation of *T*. *gondii* exposure in indigenous populations, their dogs, environment, and HPs, and fills a gap in previous studies [35]. Nonetheless, One Health concepts are present in indigenous communities' daily life and culture, particularly in Latin America, where there is intimate interaction with wildlife and natural environments, and people are highly dependent on their local ecosystems for survival [36]. As Native Americans have impacted the health and life of wild animals for thousands of years, the Columbian Exchange brought exotic species from the Old World, such as horses, cattle, sheep, and dogs, which impacted indigenous communities in a contemporary adaptive and interdependent human-animal relationship [36].

The seroprevalence of *T. gondii* IgG antibodies in the indigenous populations (225/463; 49%) was higher than that 5/50 (10%) of indigenous Cree communities (5/50; 10%) in Canada [37], lower than that of indigenous woman of Yukpa communities (93/109; 86%) in Venezuela [38], and similar to that of indigenous individuals (106/212; 50%) of East Malaysia [39]. Although IgM antibodies were observed in 11/463 (2%) indigenous people of three different communities, and in 3/168 (1%) HPs may have indicated primary *T. gondii* infection, IgM may be detected for several days, months or years after the initial infection, and remain at residual levels [19].

Our results indicate that populations living in indigenous communities lacking water sanitation were at the highest risk of toxoplasmosis. Although cat presence may be also considered a risk factor for toxoplasmosis, transmission in indigenous communities may occur by contamination of water tanks and distribution [17]. Not surprisingly, the two biggest outbreaks worldwide were reported in Brazil due to water contaminated cat feces [40,41]. Studies have shown that *T. gondii* exposure in indigenous areas in different countries could be related with their customs and culture [19,39,42,43]. As we found, these serosurveys have indicated the water consumption as an important risk factor for human toxoplasmosis. Reports of toxoplamsosis outbreaks have indicated water as the main source of transmission [43,44], associating a lack of water treament with risk of infection, particularly in areas with low socio-economic level, poor infrastructure, and precarious hygiene [18,42,45]. In the present study, seroprevalence was statistically lower in indigenous persons who have access to treated drinking water and from São Paulo State, when compared to populations ingesting water without previous treatment. Indigenous persons and dogs living in the Paraná state were more likely seropositive (17.1 and 4.1-fold odds, respectively) than those in the São Paulo state, which may be associated with available drinking water sources and the habit of drinking water directly from rivers and creeks.

In addition, both indigenous persons and 67/168 (39.9%) healthcare professional exposure was similar to that of immunocompromised people worldwide. A global metanalytic study showed that immunocompromised persons had the highest seroprevalence for IgG (48%; 31–66%) comparing to other subgroups (blood donors; childbearing age women; pregnant women; and general population) [46]. However, despite the socioeconomic vulnerability, it is not possible to argue that studied populations herein had a weakened immune system.

Although eating habits may be also an associated risk factor for Brazilian indigenous populations, water-borne outbreaks have shown higher impact on the general population outbreaks from products of animal origin [8]. Yet, indigenous communities with fish as main protein source have previously shown lower toxoplasmosis seropositivity [13]. In central-western Brazil, high *T. gondii* seroprevalence (119/148; 80.4%) was associated with water or soil oocyst contamination, as no red-meat access was verified [15]. Finally, toxoplasmosis has been mainly associated with direct ingestion of naturally contaminated water, as wild felids may live in areas with water such as creeks, ponds, lakes, and rivers [19].

In the present study, consumption of game meat was highly associated (*p* < 0.001) with *T. gondii* seropositivity among indigenous populations, as previously shown in a toxoplasmosis outbreak in an indigenous community in the Amazonia French Guiana, in which tradition of hunting and eating game meat and chicken were linked to *T. gondii* infection [43]. Moreover, other studies have also directly associated *T. gondii* infection to game meat consumption in indigenous people of Amazonia [47,48]. Another meat consumption study, based on 843 semi-structured interviews applied to students living in 28 Brazilian indigenous communities, has suggested higher consumption and preference for game meat among male students [49]. Thus, even having restricted access to commercial red-meat, the culture of hunting and eating wildlife meat should be considered in further toxoplasmosis studies, besides their ethnic eating habits. In addition, game meat as source of *T. gondii* to Brazilian indigenous may be related with the cooking type, as muscle cysts may be killed by 60 °C heating for 10 min. A limitation of this study is that game meat preparation and cooking at the point of consumption was not assessed.

We found that *T. gondii* seropositivity in indigenous persons was inversely proportional to the educational level, consistent with a previous study in which high school level or more was a protective factor against seropositivity in 276 pregnant and 124 postpartum women attended at public healthcare facilities in southeast Brazil [50]. In addition, 229 postpartum women attended in a public healthcare system of central-western Brazil with less than eight years of education had a greater chance of being seropositive to *T. gondii* (OR = 2.521) [51]. As expected, the lack of information about potential methods of transmission may have exposed vulnerable populations to infection, and may indicate the importance of education for health promotion and disease prevention.

The survey results of HPs working in indigenous communities was a major finding in this One Health approach. Despite the idea that health professionals should be safe while working in the field, no study to date has focused on such occupational risk during assistance within indigenous communities. IgG antibodies were observed in 67/168 (40%; 95% CI: 32.8–47.4) persons, similar to that observed in the general population by metanalysis (42%; 38–45%). In addition, IgM was observed only in health workers who frequently worked in indigenous communities, pointing to toxoplasmosis as an important occupational zoonotic disease. Furthermore, healthcare professional seropositivity was associated with frequency of contact and visits to the indigenous populations (*p* = 0.0001) and consumption of water from aindigenous community water source (p = 0.0001), reinforcing the role of water source as a risk factor for toxoplasmosis both for indigenous and visiting workers. We also found that age was associated with non-indigenous HPs seropositivity, as *T. gondii* seropositivity may increase with age due to increase in cumulative exposure [52].

The consumption of raw or undercooked meat was also observed as a risk for *T. gondii* exposure in non-indigenous HP (OR: 5.56; 95% CI: 1.49–21.5). Only 4/147 (2.7%) professionals reported to having meals prepared in the communities and 9/147 (6.1%) responded that they eat game meat, a variable not associated to seropositivity (*p* = 0.289). Therefore, the influence of ingestion of raw/undercooked meat for professionals might be related to an extra-laboring component. A recent meta-analysis study has shown that persons eating raw or undercooked meat had up to 3-fold more likelihood of *T. gondii* infection, regardless the animal species consumed, ranging from 0.7% to 98.3%, the variation due to individual, cultural, and religious food habits, and personal awareness [53].

Similar to their owners, dogs living in communities lacking basic sanitary systems and tap water treatment were more likely to be seropositive for toxoplasmosis (OR: 4.1). This is consistent with as other reports on dogs living under precarious hygienic conditions of two indigenous communities in the Brazilian Amazon Region [54]. In addition, we found that an outdoor lifestyle (OR: 3.84) and hunting habit (OR: 2.77) were also associated with dog seropositivity. Hunting dogs may be more exposed to *T. gondii* than dogs residing in homes due to possibly greater contact with oocysts from free-roaming cats and intermediate wildlife hosts, as described for dogs that hunt wild boars in Brazil [55]. We found ingestion of river water (OR: 2.4; 95% CI: 1.27–4.74) and owner seropositivity (OR: 37.7; 95% CI: 18.5–82.9; *p* < 0.001) were associated risk factors for toxoplasmosis in dogs, as already reported for dogs living on island and mainland seashore areas of Southern Brazil [56]. As no association between toxoplasmosis and tap or filtered water was observed in urban dogs of southeastern Brazil [57], natural water sources as rivers may pose higher exposure risk to *T. gondii* sporulated oocysts.

The presence of environmental oocysts was limited to investigation in soil samples and no specific characteristics related to soil were investigated. In only one sample (0.4%; 1/270) from an indigenous community in Paraná state was DNA from *T. gondii* oocysts detected. Contact with soil has been considered an important route for *T. gondii* transmission. As *T. gondii* oocysts are shed by the definitive host, domestic cats and wild felids, both pet cats and free-ranging stray or feral domestic cats contribute to environmental (aquatic and soil) oocyst burden and transmission of toxoplasmosis via ingestion of sporulated infective oocyst [58]. A meta-analysis reported that reasons for high prevalence rates of latent toxoplasmosis in pregnant women living in some African and South American countries could include (1) a relatively large numbers of cats and diverse *T. gondii* genotypes in these areas; (2) a lack of control measures for stray cats living in urban and peri-urban areas; and (3) high levels of environmental contamination (e.g., soil and water) and/or food with *T. gondii* oocysts [59]. For instance, in Brazil, handling the soil (OR = 2.29; 95% CI = 1.32–3.96) was considered to be a risk factor for toxoplasmosis in pregnant women [60]. Contact with soil oocysts from wild felines was reported to cause different seroprevalence among indigenous communities of northern Brazil [16]. Ability of oocysts survival in the environment is multi-faceted, and even with adequate temperatures, other variables such moisture may influence the longevity [61]. The presence of oocysts is influenced by increasing latitude (41–56°), decreasing longitude (0–40°) and increasing relative humidity (76% or more). Despite our findings, the continuing contact with soil by indigenous persons should be considered as a potential source of transmission beyond water.

Despite water samples were not collected and molecular detection was not performed herein, molecular identification of *Toxoplasma gondii* in water samples has shown limitations associated with the low oocyst amount in large volumes of water and presence of PCR inhibitors, which have biased the method standardization and prevalence studies results [62,63]. Not surprisingly, a single soil sample herein was positive by PCR for T. gondii, out of 270 (0.37%) collected samples.

Although many indigenous populations still live in naturally preserved environments, such areas have been increasingly surrounded, invaded, and destroyed by extractivism, non-sustainable agriculture, and exploratory business, leading to deterioration of indigenous economic conditions and higher health morbidity and mortality risks. Despite the interactions, accumulated knowledge and understanding, One Health has much to learnlearn from times before, as indigenous life, culture, and history have long-lived in balance with wildlife and the natural environment [36].

Finally, indigenous communities have indicated that habitants of nearby cities have constantly abandoned dogs (mostly sick) within the boundaries of indigenous communities. Although indigenous persons have a kind relationship with animals, their culture tends to consider animals as independent for their needs of shelter, water, and food. In addition, indigenous peoples commonly have wildlife pets that were mostly omnivores (eg, macaques, big birds) and herbivores such as capybaras and tapirs. As domestic dogs and cats are top of food chain hunters and carnivorous, when they prey on wildlife the results have been disastrous for pet health and wildlife conservation. Also, domestic dogs and cats require specific care and veterinary assistance. As immediate intervention, all trips to indigenous communities included vaccination, deworming, and anti-ectoparasite treatment. Also, a total of 200 vinyl banners against pet abandonment were made by the research project and posted at the entrance to 96 indigenous communities.

In summary, this study has shown untreated water sources to be a high-risk factor for infection and dogs can serve as sentinels for human toxoplasmosis, due to sharing environment and water intake. In addition, the findings herein have shown that water source should be considered as crucial for toxoplasmosis prevention in indigenous communities and always considered as associated risk factor for healthcare professionals.

## Funding

The present research was funded through the Brazilian National Council for Scientific and Technological Development-CNPq (404687/2021-0 and 401302/2022-9).

## CRediT authorship contribution statement

**Fernando Rodrigo Doline:** Formal analysis. **João Henrique Farinhas dos Santos:** Formal analysis. **Pollyanne Raysa Fernandes de Oliveira:** Formal analysis. **Nássarah Jabur Lot Rodrigues:** Formal analysis. **Rinaldo Aparecido Mota:** Methodology, Validation, Formal analysis. **Helio Langoni:** Methodology, Validation, Formal analysis. **Rogério Giuffrida:** Software, Validation, Writing – review & editing. **Vamilton Alvares Santarém:** Validation, Writing – original draft, Writing – review & editing. **Wagner Antônio Chiba de Castro:** Software, Writing – review & editing. **Andrea Pires dos Santos:** Writing – review & editing. **Louise Bach Kmetiuk:** Validation, Writing – original draft, Writing – review & editing, Supervision. **Alexander Welker Biondo:** Conceptualization, Validation, Investigation, Resources, Data curation, Writing – original draft, Writing – review & editing, Visualization, Supervision, Project administration, Funding acquisition.

## Declaration of Competing Interest

None declared.

## Data Availability

Data will be made available on request.

## Appendix A Supplementary data

Supplementary Table 1- Seroprevalence for anti-*Toxoplasma gondii* antibodies in indigenous communities in Brazil based on literature (1968 to 2019). Image 1
