# Supplementary material for: Toxoplasma gondii exposure in Brazilian indigenous populations, their dogs, environment, and healthcare professionals
Source: One Health. 2023 May 18;16:100567. doi: 10.1016/j.onehlt.2023.100567 (PMC10288134; doi:10.1016/j.onehlt.2023.100567)
Supplement: Supplementary file 1 — Supplementary Table 1- Seroprevalence for anti-Toxoplasma gondii antibodies in indigenous communities in Brazil based on literature (1968 to 2019). [file mmc1.docx]

**Supplementary Table 1**- Seroprevalence for anti-*Toxoplasma gondii* antibodies in indigenous communities in Brazil based on literature (1968 to 2019).

| **Region (State)/**  **indigenous**  **community** | **Serological analysis*** | **Seroprevalence**  **(%)** | **95% CI** | **Reference** |
| --- | --- | --- | --- | --- |
| **Central-West**  (**Mato Grosso)** |  |  |  |  |
| Xavante | IHA | 107/107 (100%) | 96.5-100 | [20] |
| Alto rio Xingu | IFAT | 131/254 (51.6%) | 45.5-57.7 | [21] |
| Kren-Akorore | IFAT/IHA | 62/70 (88.6%) | 79.0-94.1 | [22] |
| Enawenê-Nawê | IFAT/ELISA | 119/148 (80.4%) | 73.3-86.0 | [25] |
| Tiriyó | IFAT/ELISA | 310/558 (55.6%) | 51.4-60.0 | [26] |
| Waiãpi | IFAT/ELISA | 180/302 (59.6%) | 54.0-65.0 | [26] |
| Haliti-Paresí | IFAT/ELISA | 196/293 (66.9%) | 61.3-72.0 | [29] |
| **Central-West**  (**Mato Grosso do Sul)** |  |  |  |  |
| Terena | IFAT/ELISA | 67/256 (26.2%) | 21.2-31.9 | [27] |
| **North (Amazonas)** |  |  |  |  |
| Ticuna | IHA | 159/408 (39.0%) | 34.4-43.8 | [23] |
| Iauareté | IFAT/ELISA | 191/260 (73.5%) | 67.8-78.5 | [28] |
| **Southwest**  **(Minas Gerais)** |  |  |  |  |
| Xakriabá | IFAT | 27/54 (50.0%) | 37.1-62.9 | [24] |

*IFAT: Indirect fluorescent antibody test; IHA: indirect hemagglutination test, ELISA: enzyme-linked immunosorbent assay.

95% CI: Confidence Interval (calculated by the authors herein)

**Supplementary Table 2.** Locations and coordinates of indigenous communities in states of Paraná and São Paulo, including the total and sampled populations.

| **indigenous**  **community** | **Coordinates** | **Total** | **Sampled** | **%** | **Ethnicity** | **State** |
| --- | --- | --- | --- | --- | --- | --- |
| **Tekoa Pindoty** | 25°31'10.34"S 48°28'06.78"O | 40 | 22 | 55.0 | Guarani | Paraná |
| **Kuaray haxa** | 25°18'40.65"S  48°18'9.14"O | 25 | 18 | 72.0 | Guarani | Paraná |
| **Araça`í** | 25°29'23.31"S  49° 0'11.57"O | 90 | 72 | 80.0 | Guarani, Kaingang | Paraná |
|  | 25°36'56.21"S 48°56'13.05"O | 30 | 29 | 96.7 | Guarani, Kaingang | Paraná |
| **Guaviraty** | 25°36'25.35"S 48°26'36.33"O | 39 | 21 | 53.8 | Guarani | Paraná |
| **Kopenoty** | 22°15’58.20’’S 49°21’00.95’’O | 245 | 125 | 51.0 | Guarani, Kaingang, Terena | São Paulo |
| **Tereguá** | 22°15'55.31"S 49°20'53.34"O | 127 | 47 | 37.0 | Terena, Guarani | São Paulo |
| **Ekeruá** | 22°16’28.05’’S 49°22’21.95’’O | 159 | 56 | 35.2 | Guarani, Terena | São Paulo |
| **Nimuendaju** | 22°17’30.81’’S 49°22’41.03’’O | 100 | 73 | 73.0 | Guarani, Terena | São Paulo |

**Supplementary Table 3**. Seropositivity for anti-*Toxoplasma gondii* antibodies (IgG and IgM) in indigenous and healthcare professionals in indigenous communities of Southern (Paraná State) and Southeastern (São Paulo State) Brazil.

| Population | N | IgM  Seropositive (%) | IgG  Seropositive (%) |
| --- | --- | --- | --- |
| Indigenous | 463 | 11 (2.37) | 225 (48.59) |
| Healthcare professional | 168 | 3 (1.78) | 67 (39.88) |

**Supplementary Table 4**. Seropositivity for anti-*Toxoplasma gondii* antibodies (IgG and IgM) in indigenous populations (according to drinkable water supply) of Southern (Paraná State) and Southeastern (São Paulo) Brazil.

|  | **Indigenous communities with no water treatment** | | |
| --- | --- | --- | --- |
| **Indigenous**  **community** | **N** | **IgM**  **Seropositive (%)** | **IgG**  **Seropositive (%)** |
| Araça-í | 72 | 5 (6.94) | 66 (91.66) |
| Deuses da Montanha | 29 | - | 23 (79.31) |
| Guaviraty | 21 | - | 19 (90.47) |
| Kuaray haxa | 18 | 5 (27.77) | 15 (83.33) |
| Pidoty/Ilha da Cotinga | 22 | - | 18 (81.81) |
| Total | 162 | 10 (6.17) | 141 (87.03) |
|  | **Indigenous communities supplied with treated water** | | |
| **Indigenous**  **community** | **N** | **IgM**  **Seropositive (%)** | **IgG**  **Seropositive (%)** |
| Kopenoty | 125 | 1 (0.8) | 39 (31.20) |
| Tereguá | 47 | - | 19 (40.42) |
| Ekerua | 56 | - | 18 (32.14) |
| Nimuendaju | 73 | - | 8 (10.95) |
| Total | 301 | 1 (0.33) | 84 (27.90) |
| **Total of** **indigenous communities sampled** | **463** | **11 (2.37)** | **225 (48.59)** |

**Supplementary Table 5**. Seropositivity of anti-*Toxoplasma gondii* antibodies among the different categories of contact professionals in the indigenous communities of the state of Paraná and São Paulo. Being (P1) High contact level professional, (P2) Medium contact level professional, (P3) Low contact level professional.

| **Contact level** | **N** | **N IgM seropositive** | **(%) IgM**  **seropositive** | **N IgG**  **seropositive** | **(%) IgG**  **seropositive** |
| --- | --- | --- | --- | --- | --- |
| **P1** | 54 | 3 | 5.55 | 37 | 68.51 |
| **P2** | 79 | - | - | 27 | 34.17 |
| **P3** | 35 | - | - | 3 | 8.57 |
| **Total** | 168 | 3 | 1.78 | 67 | 39.88 |

**Supplementary Table 6**. Seropositivity for anti-*Toxoplasma gondii* antibodies (IgG and titers) in indigenous communities (according to basic sanitation infrastructure) of Southern (Paraná State) and Southeastern (São Paulo) Brazil.

|  | **Precarious basic sanitation infrastructure** | | | | |
| --- | --- | --- | --- | --- | --- |
|  | IgG | Seropositivity | Titer (%) | | |
| **Indigenous community** | Seropositive/  n sampled | (%) | 1:16 | 1:64 | 1:256 |
| Araça-í | 23/34 | 67.64 | 52.17 | 39.14 | 8.69 |
| Deuses da Montanha | 8/18 | 44.44 | 25 | 75 | - |
| Guaviraty | 1/8 | 12.5 | 100 | - | - |
| Kuaray haxa | 10/11 | 90.9 | 40 | 60 | - |
| Pidoty/Ilha da Cotinga | 10/15 | 66.6 | 20 | 80 | - |
| Total | 52/86 | 60.46 | - | - | - |
| **Basic sanitation infrastructure** | | | | | |
|  | IgG | Seropositivity | Titer (%) | | |
| **Indigenous community** | Seropositive/  n sampled | (%) | 1:16 | 1:64 | 1:256 |
| Kopenoty | 13/56 | 23.2 | 69.24 | 30.76 | - |
| Tereguá | 2/33 | 6.06 | 100 | - | - |
| Ekerua | 20/44 | 45.4 | 85 | 15 | - |
| Nimuendaju | 10/34 | 29.4 | 60 | 40 | - |
| Total | 45/167 | 26.94 | - | - | - |

**Supplementary Table 7.** Associated risk factors for anti-*Toxoplasma gondii* antibodies (IgG) in non-indigenous healthcare professionals in Paraná and São Paulo states, Brazil (N = 147), by univariate and multivariate statistical analysis.

|  | ELISA test result | | Univariate analysis | | Multivariate analysis | |
| --- | --- | --- | --- | --- | --- | --- |
|  | Positive (%) | Negative (%) | OR (CI 95%) | *p*-value | OR (CI 95%) | *p*-value |
| **Variables** | 54 (36.7) | 93 (63.3) |  |  |  |  |
| **Gender** |  |  |  | 0.752 |  |  |
| Female | 33 (61.1%) | 53 (57.0%) | 1 [Reference] |  |  |  |
| Male | 21 (38.9%) | 40 (43.0%) | 0.85 (0.42-1.68) |  |  |  |
| **Age (years old)** |  |  |  | 0.005 |  |  |
| 20-29 | 6 (11.1%) | 36 (38.7%) | 1 [Reference] |  |  |  |
| 30-38 | 15 (27.8%) | 18 (19.4%) | 4.82 (1.65-15.9) |  |  |  |
| 39-46 | 17 (31.5%) | 19 (20.4%) | 5.18 (1.81-16.7) |  |  |  |
| 47-65 | 16 (29.6%) | 20 (21.5%) | 4.64 (1.61-15.0) |  |  |  |
| **Ethnicity** |  |  |  | 0.760 |  |  |
| White | 5 (9.26%) | 7 (7.53%) | 1 [Reference] |  |  |  |
| Non-white | 49 (90.7%) | 86 (92.5%) | 0.79 (0.23-2.89) |  |  |  |
| frequency of contact and visits  to the indigenous populations |  |  |  | <0.001 |  |  |
| Low | 3 (5.56%) | 32 (34.4%) | 1 [Reference] |  | 1 [Reference] |  |
| Middle | 25 (46.3%) | 46 (49.5%) | 5.5 (1.72-25.6) |  | 2.59 (0.73 - 12.19) | 0.170 |
| High | 26 (48.1%) | 15 (16.1%) | 17.1 (4.98-83.7) |  | 4.74 (1.17-24.58) | 0.040 |
| **Meat consumption** |  |  |  | 0.023 |  |  |
| Well-done | 43 (79.6%) | 87 (93.5%) | 1 [Reference] |  | 1 [Reference] |  |
| Raw/undercooked | 11 (20.4%) | 6 (6.45%) | 3.63 (1.28-11.4) |  | 5.56 (1.49-21.5) | 0.011 |
| **Game meat**  **consumption** |  |  |  | 0.289 |  |  |
| No | 49 (90.7%) | 89 (95.7%) | 1 [Reference] |  |  |  |
| Yes | 5 (9.26%) | 4 (4.30%) | 2.24 (0.55-9.85) |  |  |  |
| **Water supply at**  **residence** |  |  |  | 1.0 |  |  |
| Piped water | 53 (98.1%) | 91 (97.8%) | 1 [Reference] |  |  |  |
| Artesian well | 1 (1.85%) | 2 (2.15%) | 0.91 (0.03-11.5) |  |  |  |
| **Drinking water at**  **indigenous communities** |  |  |  | <0.001 |  |  |
| No | 20 (37.0%) | 89 (95.7%) | 1 [Reference] |  | 1 [Reference] |  |
| Yes | 34 (63.0%) | 4 (4.30%) | 35.5 (12.4-133) |  | 27.0 (8.86-104.23) | < 0.001 |
| **Having meal at**  **indigenous communities** |  |  |  | 0.625 |  |  |
| No | 52 (96.3%) | 91 (97.8%) | 1 [Reference] |  |  |  |
| Yes | 2 (3.70%) | 2 (2.15%) | 1.74 (0.18-17.2) |  |  |  |
| **Hygienization of vegetables**  **at home** |  |  |  | 1.0 |  |  |
| No | 1 (1.85%) | 1 (1.08%) | 1 [Reference] |  |  |  |
| Yes | 53 (98.1%) | 92 (98.9%) | 0.58 (0.01-22.9) |  |  |  |

**Supplementary Table 8**. Retrieved *Toxoplasma gondii* oocysts from soil samples collected in indigenous indigenous communities of Southern (Paraná State) and Southeastern (São Paulo) Brazil.

|  | **Areas (positive/samples number)** | | | | |
| --- | --- | --- | --- | --- | --- |
| **State/ indigenous**  **community** | **School** | **Health center** | **Recreational area** | | **Total (%)** |
| Paraná |  |  | |  |  |
| Pidoty/Ilha da Cotinga | 0/10 | 0/10 | | 0/10 | 0/30 (0) |
| Kuaray haxa | 0/10 | 0/10 | | 0/10 | 0/30 (0) |
| Araça-í | 1/10 | 0/10 | | 0/10 | 1/30 (3.3) |
| Deuses da Montanha | 0/10 | 0/10 | | 0/10 | 0/30 (0) |
| Guaviraty | 0/10 | 0/10 | | 0/10 | 0/30 (0) |
| São Paulo | School | Health center | | Recreational area | Total |
| Kopenoty | 0/10 | 0/10 | | 0/10 | 0/30 (0) |
| Tereguá | 0/10 | 0/10 | | 0/10 | 0/30 (0) |
| Ekeruá | 0/10 | 0/10 | | 0/10 | 0/30 (0) |
| Nimuendaju | 0/10 | 0/10 | | 0/10 | 0/30 (0) |
